# Unexpected detection of clear cell sarcoma of soft tissue during single-channel endoscopic carpal tunnel release for recurrent carpal tunnel syndrome: a case report with literature review

**DOI:** 10.3389/fsurg.2026.1767446

**Published:** 2026-03-05

**Authors:** Chenxi Zhang, Shanqing Yin, Xianting Zhou, Jie Ying, Luzhe Wu, Jiadong Pan, Xin Wang

**Affiliations:** 1 Department of Hand Microsurgery and Plastic Reconstructive Surgery, Ningbo No.6 Hospital, Ningbo, Zhejiang, China; 2 Ningbo Clinical Research Center for Orthopedics, Sports Medicine & Rehabilitation, Ningbo, Zhejiang, China

**Keywords:** carpal tunnel syndrome, case report, clear cell sarcoma of soft tissue, endoscopic surgery, literature review

## Abstract

Carpal tunnel syndrome (CTS) is most commonly idiopathic or associated with wrist strain, while neoplastic compression, a rare etiology, is easily overlooked. This report describes a 50-year-old male patient presenting with numbness in the 1st to 4th fingers of the right hand. Preoperative electromyography confirmed the diagnosis of CTS, but ultrasonography showed no obvious abnormalities. During single-channel endoscopic carpal tunnel release, a protruding mass was identified at the bottom of the carpal tunnel, which was resected and sent for pathological examination. Immunohistochemical and molecular testing results indicated clear cell sarcoma of soft tissue with a maximum diameter of 4 cm. No BRAF gene V600E mutation was detected, but the EWSR1/ATF1 fusion gene was positive. Following tumor resection and multimodal adjuvant therapy, the patient achieved complete relief of numbness and maintained independent daily living activities at the 12-month follow-up, with stable pulmonary disease. This study analyzes the clinical characteristics, diagnosis, and treatment of the case, combined with a literature review. It suggests that neoplastic compression should be suspected in male patients with recurrent CTS, especially when preoperative imaging is negative, and meticulous intraoperative exploration is crucial. Literature analysis shows that wrist tumors are prone to recurrent nerve compression due to anatomical space limitations, and early identification followed by surgical resection is key to improving prognosis.

## 1 Introduction

Carpal tunnel syndrome (CTS) remains the most prevalent entrapment neuropathy worldwide (1). While primary CTS is predominantly idiopathic, the management of recurrent CTS—occurring in 3% to 19% of patients following release—presents a complex diagnostic challenge (2). Traditional clinical reasoning often attributes recurrence to benign etiologies such as incomplete release, perineural scarring, or tenosynovitis (2, 3). While existing reports of tumor-induced CTS predominantly involve benign lesions (e.g., osteochondromas, ganglion cysts, or pigmented villonodular synovitis), malignant tumors causing recurrent CTS are extremely scarce, especially in the wrist region (4). Consequently, neoplastic compression is frequently overlooked as a “blind spot” in the differential diagnosis, despite accounting for a critical minority of secondary cases.

Among space-occupying lesions, Clear Cell Sarcoma (CCS) of soft tissue is an exceptionally rare and aggressive malignancy (5). Historically termed “malignant melanoma of soft parts,” CCS typically arises from deep tendons and aponeuroses in young adults. Its indolent growth pattern and benign clinical appearance often mimic ganglions or fibromas, leading to high rates of misdiagnosis and unplanned excision (6, 7).

This case report presents an exceptionally rare and instructive presentation of CCS manifesting as recurrent CTS. The significance of reporting this case is three-fold: first, it highlights a critical diagnostic pitfall where standard preoperative ultrasonography failed to detect a 5-cm malignant mass due to its deep location and isoechogenicity. Second, it serves as an important clinical lesson to challenge the “benign inertial thinking” that often delays the diagnosis of secondary CTS in male patients. Third, by detailing the incidental intraoperative discovery and the subsequent multidisciplinary management, we aim to establish a more rigorous screening workflow—emphasizing the necessity of mandatory MRI in atypical or recurrent cases—to prevent unplanned excisions of highly aggressive malignancies.

## 2 Case presentation

### 2.1 General information

#### 2.1.1 Chief complaint

Recurrent numbness in the 1st to 4th fingers of the right hand for 6 months.

#### 2.1.2 History of present illness

The patient, a 50-year-old male, presented with a 6-month history of recurrent numbness in the 1st to 4th fingers of the right hand. The symptoms were more severe at night and in the early morning, with slight relief following activity. The patient reported no significant pain or muscle weakness at the time of admission.

#### 2.1.3 Past medical history

Four years prior to the current admission, the patient underwent open right carpal tunnel release for typical carpal tunnel syndrome (CTS). No space-occupying lesions were identified during that initial procedure, and the patient achieved complete symptomatic remission postoperatively.

### 2.2 Preoperative examinations

**Figure 1:**
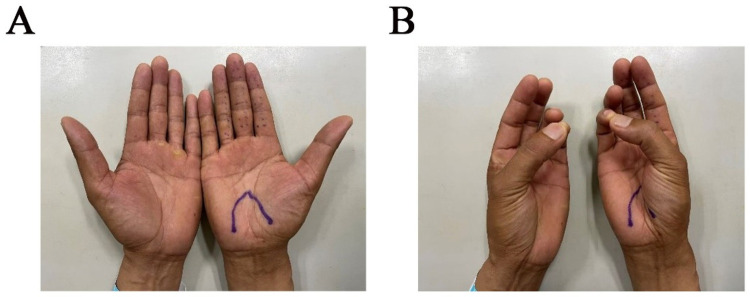
**(A)** Preoperative area of sensory numbness. **(B)** No obvious atrophy of the preoperative thenar muscles.

**Figure 2:**
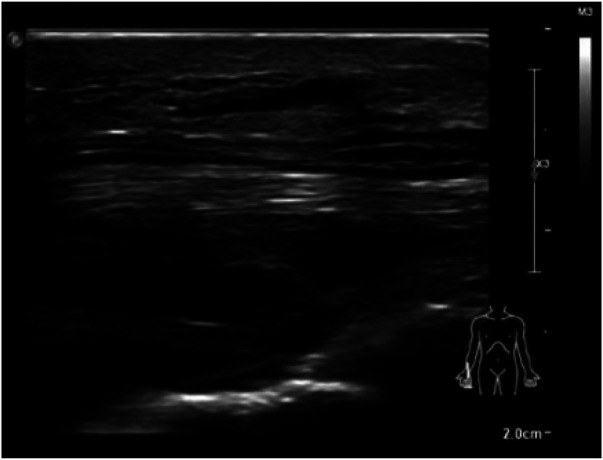
Preoperative ultrasonography examination.

Physical examination revealed hypoesthesia in the 1st to 4th fingers of the right hand, positive Tinel sign, and positive Phalen test and no obvious atrophy of the preoperative thenar muscles (Figure 1). Preoperative electromyography showed slowed conduction velocity and prolonged distal latency of the median nerve at the carpal tunnel, consistent with the diagnosis of CTS. Ultrasonography of the carpal tunnel showed no obvious space-occupying lesions or other abnormalities (Figure 2).

### 2.3 Surgical procedure

Under brachial plexus anesthesia, a single-channel endoscopic carpal tunnel release (ECTR) was performed. A 1 cm transverse incision was made at the wrist crease, localized between the palmaris longus (PL) tendon and the flexor carpi ulnaris (FCU) tendon, approximately 1 cm radial to the pisiform. After incising the thickened transverse carpal ligament, we performed a routine exploration. Upon flexing the wrist and fingers to retract the flexor tendons, an unexpected soft tissue mass was identified situated directly beneath the flexor tendons at the floor of the carpal tunnel. The mass extended from the proximal canal to the distal outlet, appearing as the primary source of neural compression.

**Figure 3:**
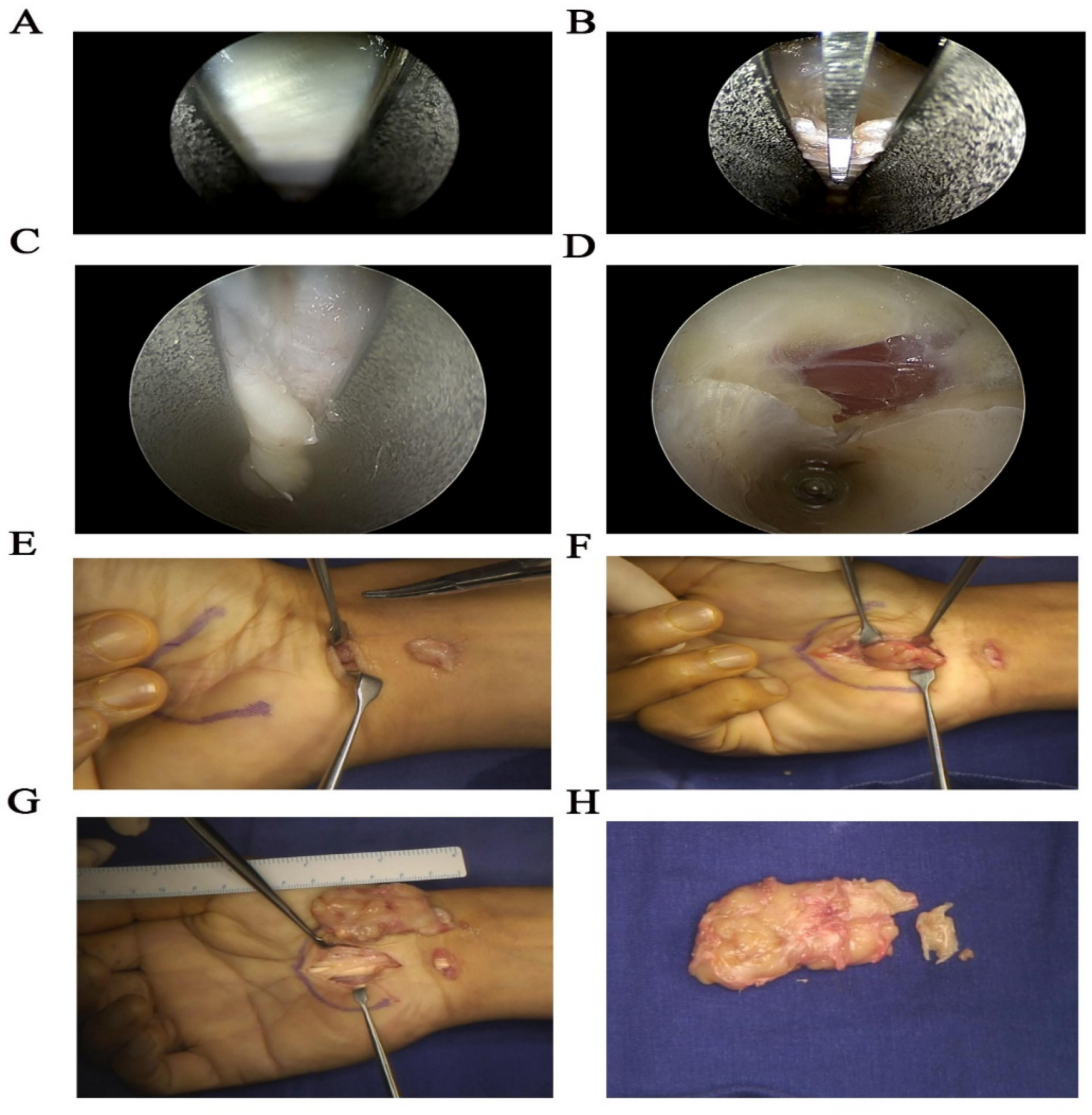
Surgical procedure. **(A)** Endoscopic view showing scar hyperplasia and compression of the transverse carpal ligament. **(B)** Endoscopic incision of the transverse carpal ligament from proximal to distal with a push knife. **(C, D)** Endoscopic confirmation of complete transection of the transverse carpal ligament. **(E–H)** Views obtained after extending the incision for open exploration and resection. **(E)** Identification of the soft tissue mass beneath the flexor tendons. **(F)** Significant adhesion between the mass and surrounding tissues. **(G,H)** Resected mass measuring approximately 5 cm × 3 cm × 2 cm.

To ensure safe resection, the original incision was extended. Intraoperative findings revealed that the median nerve was significantly flattened and displaced superficially by the underlying tumor. There was significant adhesion between the mass and the epineurium of the median nerve, as well as the surrounding deep fascia. The tumor, measuring approximately 5 cm × 3 cm × 2 cm, was meticulously dissected from the nerve and completely resected for pathological examination (Figure 3).

### 2.4 Pathological and molecular testing results

**Figure 4:**
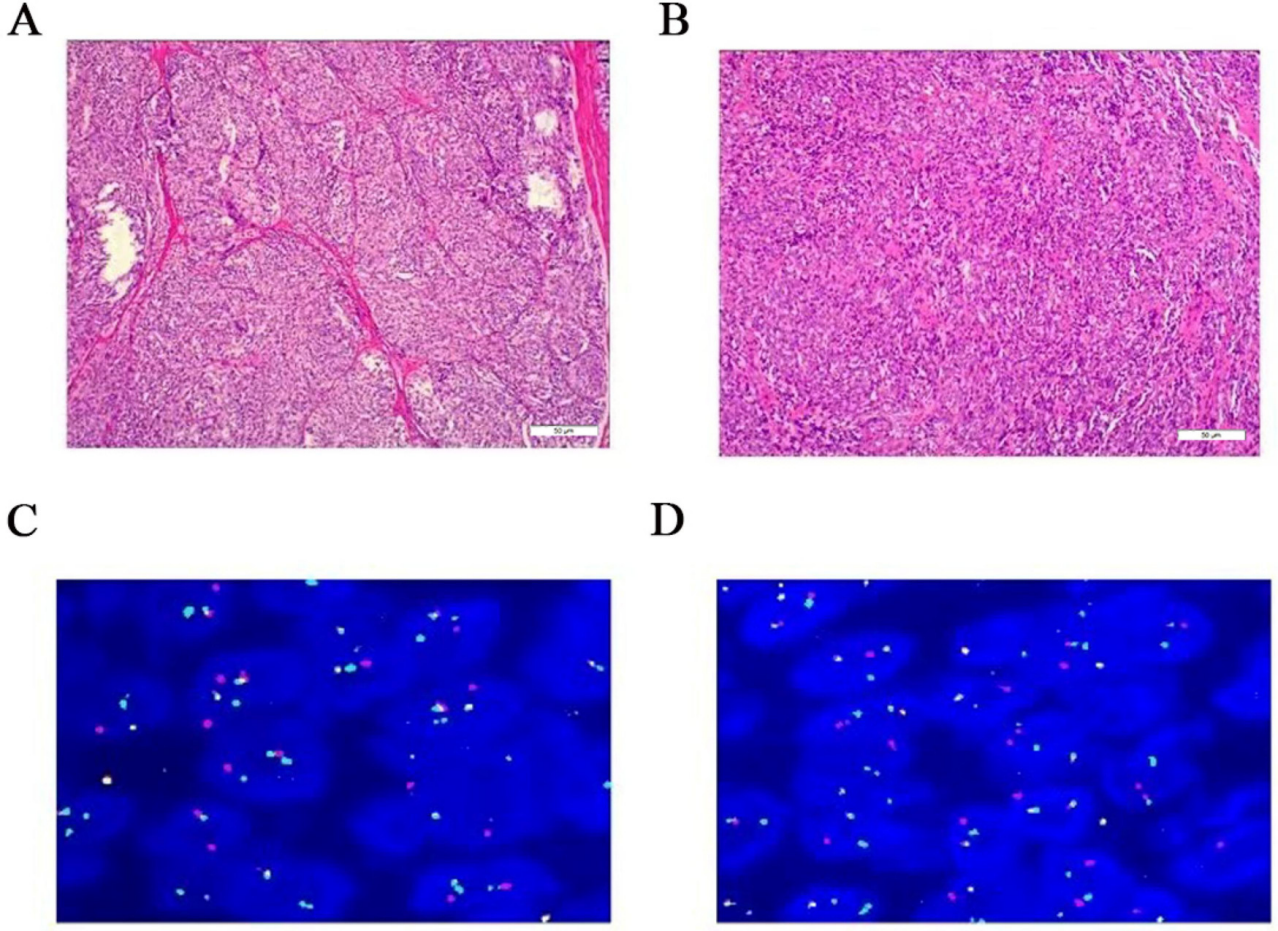
Pathological and molecular testing findings. **(A,B)** Hematoxylin and eosin (H&E) staining of the resected tissue sections showing nests of spindle-shaped tumor cells with clear or eosinophilic cytoplasm (Magnification: 100×, respectively; scale bars = 50 μm). **(C,D)** Fluorescence *in situ* hybridization (FISH) assay using an EWSR1 (22q12) dual-color break-apart probe. The presence of separated red and green signals (split signals) in interphase cells indicates a positive EWSR1 rearrangement, confirming the EWSR1/ATF1 fusion gene characteristic of clear cell sarcoma.

Molecular testing confirmed the diagnosis. The sample was negative for the BRAF V600E mutation but positive for the characteristic EWSR1/ATF1 fusion gene via fluorescence *in situ* hybridization (FISH). Detailed signal patterns supporting the translocation were identified in the analysis of 200 interphase cells (Figure 4).

### 2.5 Subsequent treatment and follow-up

Following tumor resection, the patient was administered a multidisciplinary treatment regimen. Postoperative staging was performed via chest computed tomography (CT) to screen for pulmonary metastases, which represent the most common site of metastasis for clear cell sarcoma (CCS). Given that the initial clinical suspicion pointed toward a benign recurrence of carpal tunnel syndrome (CTS), preoperative systemic imaging was not undertaken—a limitation we acknowledge in the diagnostic and therapeutic management of this case. Postoperative chest CT revealed a 14 mm × 30 mm pulmonary metastatic lesion. The patient underwent pulmonary ablation in October 2024. Systemic therapy consisted of two components:

Targeted therapy: Initially administered anlotinib, the regimen was switched to brigatinib due to suboptimal efficacy; brigatinib was subsequently discontinued because of severe rash, and the patient was switched back to anlotinib. Immunotherapy: The agent was changed from tislelizumab to sintilimab. Between April and May 2025, the patient received wrist radiotherapy with a cumulative dose of 50 Gy.

**Figure 5:**
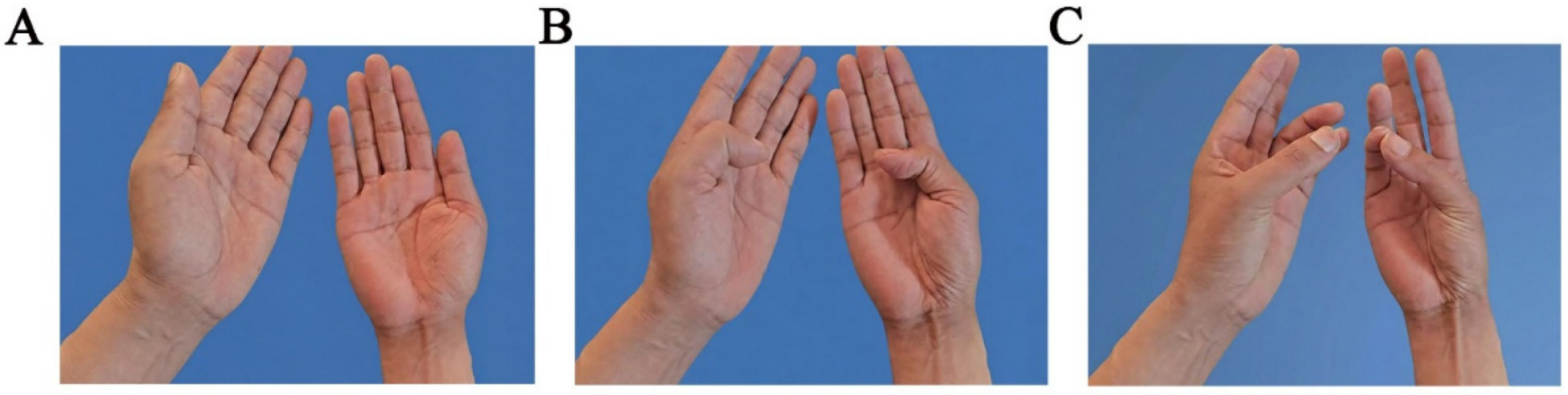
Clinical outcomes at the 1-year postoperative follow-up: **(A)** General appearance of both hands showing well-healed surgical scars at the right wrist crease, with no clinical signs of local tumor recurrence. **(B)** Close-up view of the right palm. Mild residual atrophy of the thenar muscles. **(C)** Functional assessment demonstrating successful thumb opposition to the little finger.

Regular follow-up assessments were scheduled at 3, 6, and 12 months postoperatively. Clinical outcomes documented at the 12-month follow-up visit were as follows: Wrist numbness resolved completely, and finger sensation returned to near-normal levels. Hand function improved markedly, with a CTS-6 score of 5 points (Figure 5). Serial chest CT scans confirmed that the original 14 mm × 30 mm pulmonary metastatic lesion remained stable following ablation and systemic therapy. A severe rash developed during anlotinib treatment, prompting a temporary switch to brigatinib before resuming anlotinib at a well-tolerated dose. No radiotherapy-associated complications (such as severe hand stiffness or joint contracture) were observed throughout the follow-up period.

## 3 Discussion

This study reports a rare case of soft tissue clear cell sarcoma (CCS) masquerading as recurrent carpal tunnel syndrome (CTS), characterized by a deceptive “false-negative” preoperative ultrasound and an incidental intraoperative discovery. Epidemiologically, CTS demonstrates a significant female predilection, with reported female-to-male ratios ranging from 3:1 (8–10). While diagnostic criteria (clinical symptoms and electrophysiological standards) are generally consistent across sexes, the lower prevalence of idiopathic CTS in males suggests that male gender itself should be treated as a potential indicator for secondary etiologies, such as space-occupying lesions or occupational trauma (11–13). Consequently, the recurrence of symptoms in a male patient—as presented in this 50-year-old individual—necessitates challenging the “benign inertial thinking” and warrants a heightened index of suspicion for occult pathology compared to the general female CTS population.

The management of recurrent CTS presents a complex diagnostic challenge, occurring in 3% to 19% of patients post-release (2). While traditional clinical reasoning often attributes recurrence to benign etiologies such as perineural scarring or incomplete release (2, 14–16), this case underscores the critical need to challenge “benign inertial thinking,” especially as neoplastic compression remains a frequently overlooked “blind spot” (4).

A major challenge in diagnosing neoplastic compression is imaging false negatives (17). A major challenge in this case was the “false-negative” finding on preoperative ultrasonography. Although high-frequency musculoskeletal ultrasound typically offers spatial resolution comparable to MRI, its utility was limited in this specific scenario by multiple factors. First, the tumor was located at the floor of the carpal tunnel, deep to the flexor tendons. While ultrasound visualizes superficial structures well, the overlying thickened flexor tendons and postoperative scar tissue likely caused acoustic attenuation and shadowing, obscuring the retrotendinous space. Second, the tumor morphology and tissue characteristics contributed to the diagnostic difficulty. Unlike ganglion cysts (anechoic) or schwannomas (hypoechoic with distinct boundaries), this CCS appeared isoechoic to the surrounding soft tissues. Third, as confirmed intraoperatively, the tumor was significantly adhered to the surrounding tissues. This lack of a clear fluid interface or “sliding sign” made it difficult to distinguish the neoplastic lesion from postoperative fibrosis or hypertrophic synovitis on ultrasound. Consequently, the absence of preoperative MRI constituted a significant limitation in our diagnostic process. MRI would have been superior in characterizing the tissue signal and delineating the deep anatomical boundaries (18). Therefore, we strongly advocate that for recurrent cases where ultrasound is inconclusive or inconsistent with clinical severity, MRI should be mandatory to avoid such “blind spots.”

The anatomical behavior of this 5 cm tumor offered significant insight. Intraoperative findings revealed the mass originated from deep fascia and grew anteriorly, directly displacing and flattening the median nerve against the transverse carpal ligament. This “pincer effect” explains the severity of symptoms despite the previous release. Unlike benign lesions like ganglion cysts or lipomas commonly reported in CTS (12, 19, 20), the aggressive and infiltrative nature of CCS exacerbated the nerve entrapment through both mechanical pressure and epineural adhesion.

This case emphasizes that surgeons should not limit recurrent CTS procedures to simple ligament release. Routine inspection of the carpal tunnel floor—beneath the flexor tendons—is mandatory, as space-occupying lesions are a significant cause of unilateral or atypical recurrence (21, 22). Upon detecting the unexpected mass, our shift to an extended incision and wide excision followed the established oncological principle for suspicious soft tissue malignancies (23, 24).

The management of CCS requires a shift to Multidisciplinary Team (MDT) collaboration. Given the high rate of local recurrence and systemic metastasis associated with EWSR1/ATF1 fusion-positive CCS (25, 26), surgery alone is insufficient. The integration of adjuvant radiotherapy to control local residue and targeted therapies (e.g., Anlotinib) combined with immunotherapy (Sintilimab) reflects the current paradigm in managing advanced CCS (23, 24).

## 4 Conclusion

This case demonstrates a rare instance of intracarpal clear cell sarcoma presenting as recurrent carpal tunnel syndrome. The diagnosis of this aggressive malignancy requires a multimodal approach combining pathology, immunohistochemistry, and molecular testing. Despite the diagnostic challenges posed by preoperative imaging false-negatives, a strategy of meticulous surgical resection combined with multidisciplinary adjuvant therapy can achieve favorable functional recovery and systemic disease stability.

## Data Availability

The original contributions presented in the study are included in the article/Supplementary Material, further inquiries can be directed to the corresponding author/s.
